# Conceiving reproduction in German *Naturphilosophie*. Introduction

**DOI:** 10.1007/s40656-021-00399-2

**Published:** 2021-03-30

**Authors:** Susanne Lettow

**Affiliations:** Freie Universität Berlin, Berlin, Germany

The history of scientific inquiries into reproductive processes, in particular the history of the concept of reproduction, has gained much interest during the last decades within the history and philosophy of the life sciences. Against the backdrop of the developments of reproductive technologies, genomics and synthetic biology since the mid-twentieth century – i.e. technoscientific approaches that aim at understanding and modifying generative and regenerative processes of organisms of all kinds – historicizing the notions of “heredity,” “reproduction” and “organism,” including the practices, apparatuses and infrastructures that have shaped them, is a timely endeavor.^1^ In particular, it is important to adopt a *longue durée* perspective in order to prevent oversimplistic “claims about modern innovations … based on flawed or foreshortened assumptions about the pre-modern” (Hopwood et al. 2018: 13). Thereby, it is crucial to acknowledge that any attempt to reconstruct genealogies, historical beginnings, turning points or epistemic shifts can only be written in retrospect and that historical evidence always depends on the situated knowledges of the present that shape underlying periodizations. Histories of science, as Joan Steigerwald puts it with reference to Georges Canguilhem’s notion of historical recurrence, “are inevitably informed by our present preoccupations – by extant histories of place and time, by present scientific discourses and epistemic commitments and by ongoing critical analyses of the project of science” (Steigerwald 2019: 13). This means that any history of reproduction is a history of the present in which it figures as a central technoscientific and biopolitical concept.^2^ So even if recent developments “can be traced back to the seventeenth and eighteenth centuries in Western Europe” (Stephanson et al. 2015: xvii), adopting a genealogical perspective seems to be much more promising than building on linear progressivist notions of history. Instead of searching for “origins,” clear episteme shifts or clearly defined epochs, a genealogical perspective focuses on interrelations of multiple, dispersed, sometimes even contradictory developments. François Jacob’s thesis of a “big shift from generation to reproduction” thus certainly needs “enrichment and re-examination” (Hopwood et al. 2018: 10). As Nick Hopwood argues in his exploration of the historical-semantic development of the notions of “generation” and “reproduction,” what is needed is “a larger-scale map of practices, concepts and word usage, especially for the long nineteenth century” (2018: 304).

Against this backdrop, the aim of this topical collection is to contribute to a recurrent genealogy of reproduction by focusing on the decades around 1800 and the various articulations of “reproduction” within the discursive field of German *Naturphilosophie.*^3^ Thereby the topical collection engages with ongoing research and disputes over the significance of *Naturphilosophie* for the history and philosophy of the life sciences. By focusing on the intersections of poetic, mythological, speculative and empirical knowledge claims, the contributions in this topical collection reveal the epistemic complexity and heterogeneity of the discursive field of *Naturphilosophie* that was shaped by different, albeit converging approaches. In particular, they place specific emphasis on the various articulations of sex differences. In contrast to analyses that have focused only on the emergence of a binary model of sex differences and the central status of notions of dualism and polarity, this topical collection shows that conceptualizations of reproduction were also shaped by notions of asexuality androgynity or a third sex, i.e. by multiple understandings of sex and sexual differentiation. Indeed, sex differences were not only a politically charged issue. The disputes over the question of the extent to which “reproduction” needs to be understood as a process that involves two different living beings instead of a process through which “active matter” regenerates itself were of central epistemological importance because they addressed the question of how to understand organic nature, or life – thus the very subject of “biology.” It seems that the process that Jacob has framed as transition “from generation to reproduction” involved a shift from “regeneration to reproduction,” which, too, was not a linear monolithic process. In contrast, the analyses of this topical collection reveal that during the decades around 1800 and within the relatively small field of *Naturphilosophie*, a whole range of scientific and speculative accounts co-existed that dealt with the question of how regenerative processes – for which the polyp, Buffon’s organic molecules and the infusoria were emblematic – and sexual reproduction differ and relate to each other.

## The “emergence” of Reproduction the Eighteenth Century

The term “reproduction” emerged in the disputes over epigenesis and preformation in the mid-eighteenth century.^4^ Although it was not used in a coherent way and thus did not acquire a fixed meaning, the increased usage of the term towards the end of the eighteenth century was part of a re-orientation of scientific curiosity that manifested itself in a wide range of experimental explorations of generative processes and theoretical reflections. As Staffan Müller-Wille and Hans-Jörg Rheinberger have pointed out in their seminal work on the cultural history of heredity, the conceptualization of organic reproduction “as ‘heredity’” (2007: 6) was constitutive for the emergence of what they call the “epistemic space of heredity” (ibid.: 8) in the nineteenth century. This process, they argue, relied on “an antecedent *longue durée* development in the course of which the reproduction of organic beings, in contrast to their mere production, became recognizable as a domain governed by laws of its own” (ibid.: 6). Certainly, this was not a merely conceptual development. In the course of the seventeenth and eighteenth centuries, Müller-Wille and Rheinberger stress, practices developed “by which the physical relations between organisms, instituted by individual generative acts, were ascertained, either actively by experiment or passively by keeping records” (ibid.). Maupertuis and Buffon, who contributed much to the formation of the concept of reproduction, built their theoretical reflections and speculations on experiments in animal breeding, while Lorenzo Spallanzani inquired into the secrets of animal generation through experiments in artificial insemination.^5^ Thus when the neologism “reproduction” came into use among naturalists and philosophers in the late eighteenth century, this conceptual development was embedded in a broader process of scientific explorations of the generative processes of animals and humans.

In addition, the scientific disputes and inquiries into reproduction were closely entangled with the political, economic and socio-cultural transformations of the period. In particular, the European colonial expansion that led to an unprecedented mobilization of living beings and a respective accumulation of knowledge was a structural condition of the possibility to conceptualize supra-generational reproductive processes. “Enclosing and mobilizing life on a global scale,” which made it possible to study the results of reproductive processes under the condition that living beings were separated from their endemic environments, “moved reproduction into the centre of the life sciences” (Müller-Wille 2015: 52).^6^ The botanical gardens, which “offered a space to appropriate foreign nature through acclimatization, to explore the national fauna and flora for species that could serve as substitutes for expensive imports and to set standards for national production and trade” (ibid.: 48), played a crucial role in this context. In addition, developments in animal breeding led to the new technologies “to harness and control … natural processes for economic and social reasons” (Stephanson 2015: 13).^7^

Speculations about improving the outcomes of reproduction were also geared towards humans in the context of population politics. “Population arithmetic and related probabilistic models of population began in the mid-seventeenth century” (Kreager 2018: 253), but they were “displaced by approaches prioritizing fertility from the turn of the eighteenth century” (ibid.: 254). Indeed, conceptualizing supra-individual and supra-generational processes of reproduction made it possible to calculate improvements on the level of populations. The very notion of a national population as “a self-contained entity” that has “to replenish itself” (ibid.: 266), which emerged in the context of the formation of the modern state, was closely connected to these epistemic developments. In addition, the enormous interest in all aspects of procreation and generation in the late eighteenth and early nineteenth centuries was fostered by transformations of gender, family and kinship relations, as well as by struggles over the intergenerational transmission of wealth and political privilege. As the old regime and the old patriarchal order, based on the authority of the father, vanished, new forms of kinship relations emerged. According to David Sabean and Simon Teuscher “a move from vertical to horizontal relationships, from a system distributing rights through patrilineal succession down the generations to a much more fluid set of networks constructed through marrying endogamously, mobilizing affinal kin and building obligation within ‘sibling archipelagos’” took place (Sabean et al. 2013: 13). While “all siblings were potentially equal” in this new system, it developed into “a new fraternal regime” (Engelstein 2017: 4). In this regime sisters and women more generally were addressed “as radically distinct” from men (ibid.: 5). To sum up, scientific disputes and inquiries into reproduction were thus, on multiple levels, connected with socio-economic, political and cultural transformations that challenged traditional explanations and argumentations. Through their highly speculative aspects, conceptualizations of reproduction “touched not only a sense of cosmological order” but also contributed to re-shaping social identities (Jordanova 1999: 1).

Against this backdrop, the goal of this topical collection is to shed more light on the history of “reproduction” at the turn from the eighteenth to the nineteenth century by addressing the question of how “reproduction” was conceived and articulated in *Naturphilosophie.* Thereby the collection contributes to filling a lacuna, as existing research on the relation between *Naturphilosophie* and the early life sciences only touches on the issue of reproduction without further exploring its systematic relevance. Histories of reproduction, on the other hand, have not yet systematically explored the impact of *Naturphilosophie*.^8^ The fact that the naturphilosophic approaches should be studied in the context of the history of reproduction, however, is revealed by the prominent status that the concept acquires through the writings on philosophy of nature by Friedrich Wilhelm Joseph Schelling. For Schelling, nature – which he primarily conceives of as organic – is structured through an infinite process of reproduction based on a dualism between the sexes.^9^ In the *First Outline of a System of the Philosophy of Nature*, Schelling starts from the assumption that Nature is “infinite … productive activity” (Schelling 1799/2004: 5). He argues that the endless productivity must be inhibited so that specific living entities, or “finite products” (ibid.) can appear. This, according to Schelling, presupposes the existence of two distinct sexes and every new product that results from the unification of the sexes again belongs to one of these. **“**Throughout the whole of Nature,” Schelling claims, “absolute sexlessness is nowhere demonstrable and an a priori regulative principle requires that sexual difference be taken as point of departure everywhere in organic nature” (ibid.: 36). Through the two sexes, Schelling concludes with respect to the fixity of species, the product “cannot reproduce anything but itself” (ibid.: 46) at a given stage of Nature. This means “it will reproduce itself not only as individual but simultaneously as genus to infinity (growth and procreation^10^)” (ibid.: 47). Schelling introduces his idea of the two sexes by re-articulating Johann Friedrich Blumenbach’s notion of the formative drive. According to Schelling, the formative drive “splits in opposite directions” (ibid.: 6), i.e. a male one and a female one, while Blumenbach did not make such a distinction. Most of the subsequent naturphilosophic theories that were formulated by authors such as Lorenz Oken, Henrik Steffens, Franz von Baader, Gustav Carus and Hegel referred to Schelling’s sexualized understanding of the dynamics of nature and his idea of reproduction although they modified it in various ways. Goethe, whose essay on the *Metamorphosis of Plants* (1799) was an equally important text that was read and adapted in various ways in the context of *Naturphilosophie*, developed his understanding of reproduction and sex differences while working in close contact with Schelling. As the contribution of Stefani Engelstein shows, his account of these issues, however, clearly differs from Schelling’s. Certainly, German *Naturphilosophie* contributed significantly to conceiving reproduction*,* in particular through shaping notions of sexual differentiation and impacted on the formation of the life sciences at the turn from the eighteenth to the nineteenth century.

## Naturphilosophie and the History of the Life Sciences

From the mid-nineteenth century to the second half of the twentieth century, *Naturphilosophie* had been regarded – with few exceptions^11^ – as a scientific anomaly by positivist standards. Indeed, in the years around 1800 contemporaries such as Carl Friedrich Kielmeyer already expressed critical views with respect to the scientific claims of *Naturphilosophie*. In a letter to Cuvier from December 1807, Kielmeyer “‘gladly agreed’ with Cuvier, that these investigations of natural knowledge, undertaken primarily by younger persons, have, in Germany, produced more damage than benefits” (Reill 2005: 202).^12^ In addition, Hegel launched a harsh critique of *Naturphilosophie* in the introduction to his own philosophy of nature. In the addition at the beginning of the introduction to the second part of the *Encyclopaedia*, we read that *Naturphilosophie* “made a chaotic mixture of crude empiricism and uncomprehended thoughts, of a purely capricious exercise of the imagination and the most commonplace way of reasoning by superficial analogy…” (Hegel 1970: 1). His criticism that *Naturphilosophie* is shaped through a “peculiar relationship to natural science” (ibid.: 2),^13^ however, did not prevent him from developing a philosophy of nature that deepened the epistemic gulf between philosophical speculation and the sciences (see Suzuki in this topical collection). From the mid-nineteenth century on, scientists such as Jakob Friedrich Fries and Hermann von Helmholtz then completely condemned *Naturphilosophie* as unscientific.^14^

However, in contrast to neo-Kantian and “proto-positivistic” (Beiser 2006: 8) readings, the natural philosophies of German Idealism and the Romantic period were not shaped by speculations hostile towards science and empiricism, but developed through analyses and methodological reflections on the cutting-edge natural research of the period. “All crucial philosophers of the time thought about the relation of man to nature and the possibilities of knowledge about nature to an extent not regained up to now. In addition, even if not to the same extent, naturalists around 1800 grappled directly with conceptions of *Naturphilosophie*” (von Engelhardt 1976: 1). In order to understand this close and peculiar relation between philosophy and the emerging life sciences, it is important to note that philosophy, too, acquired a new meaning and status. “In the period between the French Revolution and the opening of the new University of Berlin in 1810 there was agitation throughout the German universities for promotion of philosophy from its traditional role as a ‘lower’ preparatory faculty to that of a higher ‘scientific’ faculty on a par with law, medicine and theology” (Jardine 1996: 243). According to Nicholas Jardine, *Naturphilosophie* was “perceived as offering natural history a rise in status from a mere appendage of the medical faculty to full membership alongside mathematics, philology and physics in a higher philosophical faculty” (ibid.: 243f.). Probably the opposite also holds, i.e. that through the engagement with the scientific debates of the day, philosophy received specific attention. Certainly, *Naturphilosophie*, which built on the scientific and philosophical debates on the specificity of the organic world and in particular on Kant’s reflections on teleology, must therefore be understood as an epistemic field that indeed played a “‘strategic role’ for the birth of modern biology” (Cimino 1997: 14). This conviction is shared by Dietrich von Engelhardt, who states that “the period of metaphysical contemplation of nature in the form of romantic natural science and speculative natural philosophy was of particular significance in the modern development of the natural and especially biological sciences” (1997: 174).^15^

In particular, Schelling’s critique of the epistemic restrictions regarding the possibility of a “science” of the living that Kant had formulated in his *Critique of Judgement* was decisive for the debates concerning the epistemological problems of “biology”. Kant had claimed that such a science was impossible because the specificity of organic entities, i.e. growth, regeneration and reproduction, cannot be explained through mechanic laws while science, for Kant, was defined through mechanistic explanation. Organisms, Kant argued, are shaped through a teleological structure insofar as they are self-organized. In order to avoid physico-theological or anthropomorphic explanations, Kant added that this teleological structure was merely a “regulative” idea and did not involve a “constitutive” claim.^16^ Schelling deconstructed the distinction between the “constitutive” and the “regulative” in his philosophy of nature, which did not take the subject, the Fichtean ego, as its starting point but dynamic Nature. “Rather than attempting to ground the fundamental forces of nature in the basic activities of the transcendental ego, Schelling … insisted on placing their foundation in nature itself” (Beiser 2002: 529f.). This opened the way for naturalists to conceive of organic nature and its specific dynamics as an object of scientific inquiry and of “biology” as a science. Post-Kantian *Naturphilosophie* however was not a simple rejection of Kant’s epistemological turn but built on the unsolved questions of his philosophy and the “new space of thinking” that had been “opened by the third *Critique*” (Huneman 2006: 2).^17^

The significant role that *Naturphilosophie* played in the process of the formation of the life sciences has recently been reconstructed by John Zammito. His book *The Gestation of German Biology. Philosophy and Physiology from Stahl to Schelling* (2018) is based on the critiques of the neo-Kantian bias in the history of science and philosophy that have been formulated since the mid-1970s.^18^ A decisive attempt to understand the relation between *Naturphilosophie* and the life sciences was certainly Dietrich von Engelhard’s division of *Naturphilosophie* into “transcendental,” “metaphysical” and “scientificated” *Naturphilosophie* (1979: 105), to which Timothy Lenoir referred when he distinguished between transcendental *Naturphilosophie* and speculative and metaphysical conceptions that “removed the boundaries of possible a priori knowledge” (Lenoir 1981: 113). In Lenoir’s view, the “Göttingen School” of biology, whose members included Johann Friedrich Blumenbach, Carl Friedrich Kielmeyer, Alexander Humboldt and Gottfried Reinhold Treviranus, built on a “Kantian biological tradition.” Goethe, Lorenz Oken, Carl Gustav Carus, Schelling and Hegel, in contrast, denied, as Lenoir put it, “the claim basic to Kant’s philosophy of biology that the human faculty of understanding cannot be constitutive of organic forms” (ibid.:149). However, Lenoir’s distinction has proven to be too schematic and has been criticized by authors such as Robert J. Richards (2002) and Frederick Beiser (2002 and 2006). As Beiser puts it: there is “only a distinction in degree and not in kind between Schelling, Hegel and Novalis on the one hand and Blumenbach, Kielmeyer and Humboldt on the other hand. Any qualitative distinction underestimates not only Kant’s profound influence upon *Naturphilosophie* but also the deep tension between Kant’s regulative constraints and late eighteenth-century physiology. Even worse, it exaggerates the speculative and a priori dimension of *Naturphilosophie*, as if it had no concern with observation and experiment, while it downplays the metaphysical interests of those engaged in observation and experiment” (2006: 10). After all, Beiser emphasizes, “there was no clear distinction between philosophy and science in this period and … there was no such thing as a pure empirical science limited to only observation and experiment” (ibid.). In a similar way, Richards has claimed that “historiographic attitudes,” such as that of Lenoir, that “wish to shield the ‘real’ biologists of the period” from the “taint” of natural philosophy have “excised the heart of nineteenth-century biology” (2002: 2f.). He therefore pleas for a “reattachment” of early nineteenth-century biology “to the thought and culture that animated it” (ibid.: 3). This insight is taken up in this topical collection by focusing on conceptualizations of reproduction.

## Science and Imagination: Experiments, Analogies and the “New Mythology”

Recent publications on the interrelations of *Naturphilosophie* and Romanticism with the sciences of the day have stressed the relevance of experiments and reflections about technology in order to challenge idealist accounts and in particular the notion of organicism as an organizing intellectual principle. According to Jocelyn Holland, organicism is “perhaps the last of Romanticism’s unchallenged concepts” (2019: 30) that deserves reassessment. By analyzing the role of the lever in Romantic thought and *Naturphilosophie*, Holland questions the sharp distinction that has been drawn in intellectual history between mechanism and organicism. In fact, she shows that “the lever is deeply ingrained in early Romantic thinking, where its theory serves as a heuristic tool to model relationships between concepts, to describe processes of generation of both the individual and the universe and, more generally, as a way of addressing potential contradictions of philosophical thinking through the logic of sublation embodied by the lever in equilibrium” (ibid.). In a similar vein, Joan Steigerwald argues that “instrumental explorations of organic bodies expanded the domain of organic vitality and its boundary with the inorganic, confusing any clear delineation of the living and the nonliving” (2019: 39).

The contributions in this topical collection by Jocelyn Holland and Christine Lehleiter further explore the boundaries between the organic and the inorganic as well as the significance of experimental aspects of naturphilosophic thinking on reproduction. Holland explores how Johann Wilhelm Ritter’s reflections on rotational movements that borrowed from the alchemical tradition and evoked geological speculations “undermined polarity-based models of reproduction.” Lehleiter, in addition, shows how Goethe, in his adaptation of the *Melusine* mythos, implicitly engaged with contemporary debates about reproduction, heredity and new breeding technologies. Their analyses also reveal the central role of speculation, mysticism and literary imagination. The same holds for Stefanie Engelstein’s exploration of Goethe’s and Schelling’s references to mythology, through which both authors articulate their understandings of sexual division as well as distinctions between science and art and for Marcio Suzuki’s analysis of Hegel’s philosophical re-articulation of Goethe’s idea of metamorphosis. Indeed, the contributions of this topical collection show that *Naturphilosophie* must be understood as a discursive field that has been shaped by heterogeneous but intersecting epistemic strategies geared towards scientific, philosophical, literary, religious and mythological knowledge claims.

A standard reproach to *Naturphilosophie* that can be traced back to Hegel’s criticism is that these diverse perspectives and knowledges have been artificially drawn together through analogical thinking. Analogies, according to Hegel, do not contribute to the discovery of truth. In contrast, he argues that analogies do “not permit an inference to be made” (Hegel 1977: 152) but result in assumptions about probability only. In fact, this purely negative account of analogical thinking is questionable and the use of analogies, metaphors and comparisons has been widely discussed in contemporary philosophy of science. With regard to the “common ground between literature and science” that existed in the early nineteenth century prior to the development of the so-called “two cultures,” Devin Griffiths has introduced the notion of “harmonic analogies” in order to explain the epistemic productivity of analogies. “Analogies,” he argues, “give voice to patterns that have no name” (2016: 11) and thus play an important role in the process of discovering new problematics. In contrast to “formal analogies” through which “a previously understood pattern of relationships is applied to a new context,” harmonic analogies explore “a pattern between two different sets of relationships” (ibid.: 18). These harmonic analogies are highly creative because they “allow significant shared features to emerge through contact between two different domains placed in serial relation” (ibid.). However, with regard to the epistemic strategies of *Naturphilosophie* the question arises as to whether the problem is correctly framed when reducing it to the use of analogies. In particular, it seems crucial to take into account – as several contributions to this topical collection do – the call for a “new mythology” that shaped these strategies. As Manfred Frank has shown, the idea of a new mythology first emerged in the context of post-Enlightenment debates about the possible meaning of ancient mythologies in modernity. What was at stake in these debates was a critique of analytic rationality, although the “new” mythology was understood as a mythology of reason. This means that ít was by no means a plea for irrationalism, pure speculation or a return to theology but a “meta-critique of enlightened critique” (Frank 1982: 188) based on a dialectical understanding of reason which asserts its own limitations and legitimizes itself by providing its own epistemic and ethical foundations. Scientific rationality, in this context, was an issue of concern, so that the boundaries between science and art, in particular poetry, or between reason and imagination, were constantly negotiated and re-negotiated.

It is this amalgamation of different epistemic strategies through which *Naturphilosophie* was constituted in the early nineteenth century that – despite later historiography – contributed immensely to the development of biology. In contrast to attempts that merely aim at re-integrating *Naturphilosophie* into the history of the life sciences and that highlight its impact on the scientific developments that have occurred from the nineteenth century onwards, it is of central importance to take seriously the interrelations of scientific, poetic, mythological, religious and philosophical references. Re-integrating *Naturphilosophie* into the history of the life sciences then means also to historicize the boundaries through which the epistemic object of the history of the life sciences has been shaped and to challenge the boundaries of this field of knowledge. Indeed, when history of science itself emerged as a distinct field in the twentieth century, it built on an understanding of science and scientific disciplines that is anachronistic with regard to the decades around 1800. Taking *Naturphilosophie* seriously thus means to scrutinize the historically contingent relations between philosophy, science and history of science in order to establish new, productive perspectives on this complicated relationship. A central aspect in this respect is the nexus between “mythology” and reason, or the imaginative and cultural-symbolic excess of the sciences as they were constituted and separated from other modes of knowing during the nineteenth century. This excess is particularly powerful when it comes to scientific and technological explorations of “reproduction” and public disputes over the use of human stem cells, germ line experimentation or the invention of new artificial reproduction technologies (ART). Far from being results of “neutral” science, these endeavors are heavily charged with and evoke various biopolitical imaginaries and imaginations about what kinship, family and identity mean.

With regard to the imaginative aspects of science, interdisciplinary research on the intersections of science, literature and the arts has added important insights to the field of history and philosophy of the life sciences. Although, as Christine Lehleiter has stated, there have been “hesitations and delays in establishing and institutionalizing the field” (2016: 2), inquiries into the interrelations between literature and science have now flourished at least since the 1990s. These do not concern only the transposition of scientific problems and developments into literature or narrative structures that shape scientific texts. In addition, research on “literature and science” has shown how porous the boundaries between the so-called “two cultures” have always been, certainly in the period around 1800. The significance of Goethe’s scientific writings, for example, had certainly been acknowledged for a long time and the idea that his notion of metamorphosis was of utmost theoretical importance for Schelling and subsequent *Naturphilosophie* is widely dicussed now.^19^ In addition, scholarly attention to his non-scientific writings, too, provides crucial insights into the history of the life sciences, as Lehleiter argues.

Some of the contributions in this topical collection are part of the field of “literature and science,” others are mainly indebted to the history of philosophy and history of science. Since the contributions all converge with regard to the authors they discuss and with regard to the topics of reproduction and sexual differentiation the collection provides an example of how these different disciplinary approaches can intersect.

In her contribution *De-polarizing Reproduction: Two Cases from Naturphilosophie, circa 1800*, Jocelyn Holland scrutinizes how the Romantic physicist Johann Wilhelm Ritter connected the notion of reproduction to rotational movement. She analyzes how Ritter, who heavily builds on the alchemical tradition for conceiving generative capacities, complicates binary models of sex differences. Nonetheless Ritter, as Holland shows, also integrates contemporary scientific ideas about centrifugal and gravitational forces and introduces the androgyne figure of a “third sex.” Stefani Engelstein also argues that the binary understanding of sexual reproduction that structures Schelling’s philosophy of nature was not the only theoretical option available. In particular, Schelling’s dualistic account clearly differed from Goethe’s more nuanced understanding of sexual reproduction. Engelstein’s analysis focuses on Goethe’s and Schelling’s references to “mythology” through which they negotiate the relation between poetry and science or science and philosophy. Goethe’s attitude towards sexuality, however, is more complex than Schelling’s in so far as sexual reproduction, for Goethe, only emerges at a certain stage of organic development but is not – as for Schelling – a metaphysical condition of organic development. So, while Goethe also conceives of non-sexualized modes of generativity, Schelling introduces a binary model that is at the same time hierarchic, as Engelstein shows in her exploration of Schelling’s theoretical engagement with mythology and his devaluation of the female. Gregory Rupik’s analysis of Goethe’s exposition of plants’ transformations in *The Metamorphosis of Plants* supports this conclusion. He shows how Goethe departed of from Linnaeus’ and Christian Wolff’s understandings of teleology and introduced an a-teleological understanding of propagation by building on insights that he found in Herder’s philosophy of nature. In particular, Rupik argues that Goethe conceived of nature as “active productivity” and of sexual propagation as not being different in kind from the process of metamorphosis that shapes the plant through each moment of its life. So, despite Goethe’s close collaboration with Schelling and the deep impact that his notion of metamorphosis had on many *Naturphilosph*s, the way Goethe conceived of procreative and generative processes seems to be much less static. In addition, Christine Lehleiter’s analysis of Goethe’s adaptation of the myth of *Melusine* – a narrative which centers on the union between a male human and a supra-natural female – reveals that Goethe did not only engage with the contemporary scientific theories and disputes over growth, regeneration and reproduction and the status of sex differences within the realm of the organic. Moreover, Lehleiter analyzes how Goethe evaluated contemporary debates on heredity and breeding. In his alteration of the mythical narrative, Lehleiter argues, Goethe refers to contemporary scientific debates and new developments in animal husbandry. Although Goethe, in his scientific writings, did not consider sexual reproduction as a driver of change or species transformation so that organic variation was clearly defined to the unchangeable boundaries of the species, Lehleiter shows that in his *New Melusine*, Goethe arrives at different conclusions. The story not only suggests that “novelty beyond variation” is possible, but it can also be understood, as Lehleiter suggests, as an early reflection on biopolitical projects of human breeding. Marcio Suzuki, in his chapter on *Reproduction versus Metamorphosis: Hegel and the Evolutionary Thinking of His Time* finally reconstructs Hegel’s distinction between Nature and Concept that allows him to distance himself from all those naturalists and philosophers who indeed (like Goethe) took into consideration the idea that species and other organic boundaries might not be fixed but subject to transformation. With reference to Goethe’s concept of *metamorphosis* Hegel claims that only “the Concept” – and thus not Nature – is capable of developing something new. Suzuki shows how Hegel engages with theories of reproduction from Leibniz and Buffon to Schelling and develops his own theoretical account of sexual reproduction. However, through his re-articulation of *metamorphosis* or development as a philosophical-conceptual process he introduced a separation between philosophy and science that, in the decades that followed, became extremely powerful.

The initial idea for this topical collection goes back to the workshop “Conceiving Reproduction. The Impact of German *Naturphilosophie*”, organized by Susanne Lettow and Gregory Rupik, that took place at Freie Universität Berlin on July 6–7, 2018. The workshop and this publication have been funded by the German Research Foundation DFG as part of the research project “Genealogy and Belonging. Reproduction, Descent and Kinship in Post-Kantian *Naturphilosophie*”. I thank the anonymous reviewers of this project as well as the reviewers who have contributed to this topical collection for their support. Special thanks go to Gregory Rupik for co-editing the topical collection and to Florence Vienne for comments on an early draft of this introduction.

## Footnotes

1 Cf. for example Steigerwald 2019; Cécilia Bognon Küss 2019; Hopwood et al. 2018; Stephanson et al. 2015; Bock von Wülfingen et al. 2015; Holland 2009; Müller-Wille et al. 2007a, 2007b.

2 As Nick Hopwood, Rebecca Flemming and Lauren Kassell highlight in the introduction to the volume *Reproduction. Antiquity to the Present*, “frameworks for the history of reproduction were built in encounters between critical political and intellectual agendas, particularly within feminism and pre-existing disciplines. Histories of medicine, demography, the family … supplied foundations, component parts and narratives to critique” (2018: 5).

3 As Canguilhem stresses, the genealogy of scientific concepts focuses on discontinuity instead of construing linear developments (Canguilhem 1968). Foucault has built on this and his understanding of recurrence in the *Archaeology of Knowlege* (1969) differs significantly from his earlier idea of episteme shifts. “*Recurrent distributions* reveal several pasts, several forms of connexion, several hierarchies of importance, several networks of determination, several teleologies, for one and the same science, as its present undergoes change” (Foucault, quoted in Steigewald 2019: 13). The concept of “recurrence” was first introduced by Bachelard, who claimed that, in the words of Hans-Jörg Rheinberger, the sciences “constantly trangress themselves but remain recursively, in the sense of a historical linkage, related to their inventory of problems” (Rheinberger 2007: 45f., *my translation*).

4 On the history of the different usages of the terms “generation” and “reproduction,” and the German words *Fortpflanzung* and *Zeugung* cf. Hopwood 2018, Holland (2014), Lettow (2014).

5 Cf. Terrall 2007 and Orland 2017.

6 Müller-Wille argues that Linné’s natural history was a main driver of this process and that the oppostion between his “static” taxonomic system and the temporalization of nature in the late eighteenth century does not hold.

7 “Early modern breeders aimed to arrest degeneration. Practical and cosmological frameworks encouraged breeding for the status quo. (…) The paradigm shifted when breeder’s objectives turned towards ‘ideal types’ designed by humans” (Wilmot 2018: 398). According to Sara Wilmot, the new “apparatus of elite breeding … came into being in the eighteenth century” (ibid.). On articulations of human breeding and the emerging race discourse cf. Lettow 2015.

8 Peter Hanns Reill (2005) and Philippe Huneman (2006) however make clear that issues of generation, gender and sexuality play a strategic role in *Naturphilosophie*. For Reill the naturphilosophic explanations of gender and sexuality express most clearly “the hierarchic and dynamic-static element” (2005: 220) and Huneman stresses that “the peculiar status of sexuality is a general feature” (2006: 16) of Naturphilosophie.

9 Cf. also Alison Stone 2015, Lettow 2013.

10 Keith R. Peterson translates the German word “Zeugung” as “reproduction,” which is misleading because Schelling here refers to “Zeugung” (procreation) as an aspect of what he conceives reproduction to be.

11 In 1941, Alexander Gode-von Aesch published the book *Natural Science in German Romanticism*, in which he argued that science and literature “are to be regarded, not as separate, tangential fields of expression, but as two aspects, identical in physiognomy, of the general movement that marked Germany’s cultural life in the transition from the Eighteenth to the Nineteenth century” (1941: 12).

12 Kielmeyer’s own relation to *Naturphilosophie*, however, is complex. His address *On the Relations Between the Organic Forces* from 1793 was enthusiastically received by the *Naturphilosophs* and Schelling even claimed that with Kielmeyer “an entirely new epoch of natural history” had begun (quoted by Zammito 2018: 245). Zammito concludes that in the first years of the new century “Kielmeyer may well have begun to rue the impulses he had helped to set loose in life sciences, paleontology and philosophy of nature” (ibid.: 265).

13 “The category ‘science’ itself … is a relative historical novelty. In Europe’s various languages, the word took on its modern meaning only over the course of the nineteenth century. Before 1800, there was no standard collective term for the sciences of nature taken together” (Phillips 2012: 3). The German term *Naturwissenschaft* (natural science) had its first entry in the Brockhaus lexicon in 1824 (ibid.: 28).

14 The neo-Kantian turn in science and philosophy in the second half of the nineteenth century in German academia impacted greatly on the development of logical empricism in the twentieth century (Friedman et al. 2006) and on the respective strands in the history and philosophy of science that dominated these fields until the 1970s and partly continue to dominate.

15 This also holds with regard to the institutional aspects. “Most leading figures in German Romantic science were tirelessly social. Henrik Steffens led the *Natural Scientific Section* of Breslau’s *Silesian Society for the Culture of the Fatherland* (*Schlesische Gesellschaft für vaterländische Cultur*). Carl Gustav Carus played a prominent role in the local scientific societies of his native Dresden. Lorenz Oken, famously, was the guiding force behind the *Society of German Natural Researchers and Doctors* (*Deutsche Gesellschaft der Naturforscher und Ärzte*). His fellow *Naturphilosoph* Christian Nees von Esenbeck initiated an effort to revive the *Leopoldina*, the official academy of the now-defunct Holy Roman Empire” (Phillips 2012: 87).

16 Indeed, this distinction gave rise to a wide range of interpretations and criticisms. “It might appear,” as Philipp Huneman puts it, “that in the end Kant was quite alone in insisting on the sharp distinction between regulative and constitutive and mainly for metaphysical reasons” (2006: 8).

17 Certainly, this does not mean, as Amanda Jo Goldstein criticizes, that Kant “discerned and conceptualized the *organism* as the special object of the then-emergent sciences of life” (Goldstein 2018: 4) and that he identified “the epistemological gounds of modern biology” (ibid.). Such a view completely neglects the post-Kantian re-articulations of his understandings of science, nature and organism as well as developments beyond the German context.

18 Cf. for example Andrew Cunningham, Nicholas Jardine (ed.): *Romanticism and the Sciences*. Cambridge: Cambridge University Press, 1990. They state that “the past fifteen years have … seen a revival of scholarly interest and a series of reassessments” (1990: xix) of Romanticism.

19 Cf. Christine Lehleiter (2015) and Stefani Engelstein (2008). On the collaboration between Goethe and Schelling cf. Nassar (2010).
